# A contrastive learning method integrating pathological prior information for effective differentiation of histological categories in lung squamous cell carcinoma

**DOI:** 10.1186/s12885-025-15459-0

**Published:** 2025-12-17

**Authors:** Mingci Huang, Weijin Xiao, Gen Lin, Chao Li, Haipeng Xu, Yunjian Huang, Shengjia Chen, Chuanben Chen, Yang Sun, Qiaofeng Zhong

**Affiliations:** 1 Department of Medical Oncology, Clinical Oncology School of Fujian Medical University, Fujian Cancer Hospital, Fuzhou, 350014 China; 2 Interdisciplinary Institute for Medical Engineering, Fuzhou University, Fujian Cancer Hospital, Fuzhou, 350116 China; 3 Department of Pathology, Clinical Oncology School of Fujian Medical University, Fujian Cancer Hospital, Fuzhou, 350014 China; 4 Department of Radiation, Clinical Oncology School of Fujian Medical University, Fujian Cancer Hospital, Fuzhou, 350014 China; 5 Department of Gynecology, Clinical Oncology School of Fujian Medical University, Fujian Cancer Hospital, Fuzhou, 350014 China; 6 Department of Medical Epidemiology and Biostatistics, Karolinska Institutet, Stockholm, Sweden

**Keywords:** Self-supervised learning (SSL), Sample-positive (SP) technique, Lung squamous cell carcinoma (LSCC), Whole slide images (WSI), Contrastive learning, Pathological prior information

## Abstract

**Background:**

Advancements in digital pathology and computer technology have spurred artificial intelligence in histopathology, but the complexity of whole slide images (WSIs) poses challenges for manual annotation and traditional supervised learning.

**Methods:**

We propose the Sample-Positive (SP) technique, which utilizes adjacent tissue morphology in WSIs to effectively sample positive examples. By integrating pathological prior information that reflects spatial adjacency of similar tissues with self-supervised learning (SSL) frameworks like SimCLR, MoCo-v3, and SinCLR, we developed an SSL method for WSI. We validated this approach on a dataset of 65 lung squamous cell carcinoma (LSCC) cases, covering four histological categories: necrosis, tumor, stroma, and epithelium. Performance was benchmarked against supervised models and original SSL frameworks using fine-tuning and linear evaluation, with metrics including accuracy (Acc), AUC, and F1 score.

**Results:**

Our proposed SP technique outperformed baseline SSL methods in fine-tuning and linear evaluation tasks on the LSCC dataset. SPSimCLR and SPMoCo-v3 achieved the highest F1 scores, with SPSimCLR (0.9132) showing a 0.7% improvement over SimCLR (0.9067) and SPMoCo-v3 (0.9133) a 0.5% improvement over MoCo-v3 (0.9088) in fine-tuning, and SinCLR (0.9074) performs comparably to the original SSL methods. In linear evaluation, SPSimCLR (0.9082) improved F1 scores by 1.0% over SimCLR (0.8978), and SPMoCo-v3 (0.9060) improved by 1.2% over MoCo-v3 (0.8942), and SinCLR(0.9021) is surpass the original SSL methods. Ablation studies revealed that overlapping sampling slightly outperformed non-overlapping sampling, and that models trained on patches with single tissue types performed better than those trained on patches containing multiple tissue types.

**Conclusions:**

Overall, combining the SP technique with contrastive learning shows significant improvements in distinguishing histological categories in LSCC, making it effective for WSIs of non-diffuse cancers.

**Supplementary Information:**

The online version contains supplementary material available at 10.1186/s12885-025-15459-0.

## Introduction

Histopathological slide analysis is the gold standard for cancer diagnosis [1, 2]. While digital pathology scanners have propelled the development of computational pathology, computer vision has achieved remarkable success across various domains, prompting its integration with pathology analysis [3]. However, whole slide images (WSIs) typically have resolutions exceeding 40,000 × 40,000 pixels, classifying them as ultra-high-resolution images [4]. Traditional computer vision methods, designed for smaller pixel images due to the properties of natural images and hardware limitations, face challenges when directly applied to WSIs [5]. Although downsampling WSIs can meet the training requirements of traditional computer vision models, excessive downsampling results in the loss of cellular-level information, thereby compromising the accuracy of pathological analysis.

To address the issues mentioned above, a common approach involves dividing the WSIs into smaller patches using a sliding window, making them more suitable for computer vision models [6–8]. These patches are labeled based on annotations from pathologists, and a patch feature extractor is trained on the annotated dataset. Once trained, the feature extractor is used to derive features from all the patches within the WSIs. These patch features are then aggregated using an algorithm to infer the label of the WSIs. However, a major limitation of this method is the challenge of obtaining precise and consistent annotations.

The fine differences in pathological tissue morphology can result in significant errors when annotations are made at low magnification. While accurate annotation requires high magnification, the enormous number of pixels in WSIs makes this process extremely labor-intensive, costly, and prone to errors. Moreover, different pathologists may interpret and annotate the same image differently, resulting in inconsistencies. The varying quality of these annotations can negatively impact the model’s learning process, ultimately affecting the accuracy of pathological slide analysis [9].

Weakly supervised learning, which does not require precise annotations, is commonly used when labeled data is insufficient or expensive [10, 11]. Due to the high cost and inconsistency of WSIs annotations, weakly supervised methods have gained significant attention as a solution. In the context of pathological images, weakly supervised learning allows pathologists to provide general WSIs labels after a brief review, greatly reducing the complexity and cost of annotation.

Multiple Instance Learning (MIL) is the most widely used weakly supervised technique in the field of pathological image analysis [12–15]. In MIL, each WSI is represented as a “bag” or “set of instance” rather than a single sample, with each instance corresponding to a patch. A bag is labeled as positive if at least one instance within it is positive; otherwise, it is labeled as negative. Although MIL helps reduce annotation costs, it has certain limitations, such as the tendency to overlook contextual information in WSIs and the difficulty in effectively analyzing fine-grained tissue structures. To address these limitations, researchers have proposed methods like CLAM [14], an attention-based MIL approach. CLAM leverages attention mechanisms to automatically identify diagnostically significant subregions and generate relevant heatmaps. However, these heatmaps still have limitations, such as coarse annotations, and the model’s performance heavily depends on the quality of the feature extractor. As a result, there has been a growing interest in the field of self-supervised learning (SSL).

SSL is a technique that does not rely on labeled data; instead, it trains models by leveraging the inherent structure within the data itself to develop robust feature extraction capabilities. This approach makes SSL highly adaptable to various downstream tasks and holds considerable promise for broad applications. Currently, SSL methods are rapidly closing the performance gap with supervised learning techniques. Since SSL generally requires large datas, and WSIs can be segmented into numerous patches using a sliding window approach, this method is well-suited to the needs of SSL. Therefore, SSL has emerged as an effective strategy for training feature extractors in pathological image analysis [16, 17]. Feature extractors trained through SSL can be employed in multiple instance learning to extract and aggregate representations for slide label prediction, as well as to perform detailed analysis of individual patches.

Contrastive self-supervised learning is a prominent paradigm within the SSL framework. Contrastive learning is based on the consistency assumption, which posits that positive sample pairs should exhibit similar feature representations, while negative sample pairs should demonstrate dissimilar feature representations. Based on this assumption, contrastive learning optimizes sample features by bringing positive samples closer together and pushing negative samples apart.

Typically, contrastive learning uses different augmentations of the same image as positive sample pairs and treats augmentations from different images as negative sample pairs. However, when applied to WSIs, this approach faces several unique challenges. First, the number of tissue categories in WSIs is limited, and adjacent patches often exhibit highly similar morphological features; for example, the texture and structure within the same tumor or stroma region are almost identical. If these adjacent patches are simply treated as negative samples, the model may incorrectly separate their representations, disrupting the clustering structure of patches from the same tissue type. Furthermore, WSIs are extremely large, with each slide containing tens of thousands of patches, many of which may belong to the same tissue type. In this case, randomly sampled negative examples are likely to include a large number of patches from the same class, further blurring the definition of positive and negative samples. Therefore, traditional contrastive learning is prone to the problem of “separating patches from the same tissue type” when applied to WSIs. Moreover, a significant drawback of contrastive learning is its reliance on a large number of negative samples to facilitate learning, which necessitates substantial memory resources.

To reduce the reliance on negative samples, knowledge distillation-based methods have been proposed, which focus solely on positive samples, such as augmented versions of the same image, and do not require negative samples. However, by focusing only on positive samples, these methods might overlook global sample information, posing a limitation as well.

To address the issues mentioned above, we propose a method called Sample-Positive (SP), which incorporates pathological prior knowledge that reflects spatial adjacency of similar tissues by sampling multiple patches with the same label within pathology images. In existing contrastive learning frameworks, positive samples are typically obtained through two different augmentations of the same image. By introducing the SP method, patches sampled from the same large image block are treated as positive samples, providing an effective source of positives for contrastive learning. This approach significantly overcomes the limitations of contrastive learning in the WSI domain while retaining its ability to capture global information. Our experiments demonstrate that SSL can enhance the model’s representational capacity, and introducing the SP technique within the contrastive learning paradigm can further improve the performance of contrastive learning models.

Our contribution can be summarized as follows:

1. We propose the SP technique, which leverages prior information that spatial adjacency of similar tissues from pathological images to enhance contrastive learning methods and achieve better performance.
2. Building on the SP technique, we introduce SinCLR, a framework that requires only a single augmentation to perform self-supervised learning, effectively reducing memory requirements and improving training speed.
3. We compare the performance of various SSL frameworks and demonstrate their practical utility in the field of pathological image analysis.

## Related Works

### Self-supervised learning

Self-supervised learning (SSL) is a method that learns sample representations without relying on manual labels and has been widely applied in the field of computer vision [18, 19]. SSL methods utilize the inherent structure and consistency of the data itself as supervision signals, learning robust representations by maximizing the information density of the data. This approach enhances the model’s generalization capability and performance. Several paradigms have emerged, including contrastive-based methods and knowledge distillation-based methods. Typically, these techniques generate augmented views from individual data instances and train the model to generate similar outputs for each view.

Contrastive learning is an important method in SSL, aimed at obtaining informative and concise representations from unstructured data by bringing positive sample pairs closer together and pushing negative sample pairs further apart. Several contrastive learning-based methods have been proposed, including CPC [20], SimCLR [21], and MoCo [22]. CPC is a foundational work in self-supervised contrastive learning, introducing the InfoNCE loss and providing inspiration for subsequent methods. SimCLR and MoCo are two concise and widely adopted approaches in the field of contrastive learning. Both methods generate positive sample pairs by applying different augmentations to the same image and create negative sample pairs by pairing augmentations from different images. They employ the infoNCE loss as the optimization objective and utilize a dual-branch structure. MoCo differs by using two networks: an online network for feature extraction and a fixed target network for preserving historical features. The parameters of the online network are updated through a momentum-based approach, which combines them with the parameters of the target network. In contrast, SimCLR uses a dual-branch structure with the same network for feature extraction, and its network parameters are updated exclusively through back propagation.

Unlike contrastive learning, knowledge distillation-based methods rely only on positive sample pairs and do not require negative sample pairs. As a result, they do not necessitate large batch sizes for training. Representative methods in this category include BYOL [23] and SimSiam [24]. BYOL utilizes a momentum update mechanism to establish a target network by applying exponential moving averages to the weights of the online network. It trains these two networks using random data augmentation and minimizes the distance between the outputs of the online network and the target network to learn high-quality representations. SimSiam, influenced by BYOL, simplifies the momentum update mechanism and adopts a symmetric architecture. It uses a dual-branch architecture with the same network, where each branch predicts the output of the other. To ensure meaningful representations, SimSiam utilizes stop-gradient operations.

### Supervised contrastive learning

Prannay Khosla et al. [25] enhanced contrastive learning by incorporating label information, developing a method known as supervised contrastive learning. The foundation of contrastive learning is the consistency assumption. However, due to the lack of sample labels, traditional contrastive learning only treats augmented samples of the same data as positive pairs and considers all other samples as negative pairs. This setup can potentially separate representations of samples from the same class. They believe that in data with known label information, contrastive learning can fully utilize the label information by treating augmentations of samples from the same class as positive samples and samples from other classes as negative samples. Consequently, they proposed a supervised contrastive learning (SupCon) loss that builds on traditional contrastive learning by incorporating label information. This loss function is better at focusing on challenging positive and negative sample distinctions, leading to improved performance and ensuring that normalized embeddings from the same class are more closely aligned than those from different classes.

### Self-Supervised learning in pathology

Histopathological images are characterized by high resolution, making it challenging to obtain large volumes of accurately annotated data [26]. SSL, which does not require annotated information, has proven effective in addressing these challenges. Many current studies employ SSL to extract features from WSIs and apply these features for downstream analysis in histopathology, leading to notable advancements in the field [27].

Chen and colleagues [28] developed an end-to-end multimodal fusion framework designed for survival prognosis prediction, leveraging both histopathological images and genomic data, and initializing the model with the CPC self-supervised method. In another study, Ciga and collaborators [29] provided extensive experimental evidence showing that self-supervised pretraining methods can yield improved features, thus enhancing the performance of several downstream tasks. They found that the effectiveness of contrastive self-supervised pretraining is more influenced by the diversity of the unlabeled training set than by the number of images.

These studies highlight the potential of SSL to enhance feature extraction and its applicability to pathology image tasks when labeled data is limited. Building on existing SSL frameworks, we explore SSL methods more suitable for pathology. Based on observations of histopathological images, we propose the SP method, which increases the number of positive sample pairs by sampling from large pathological image regions. This approach is conceptually similar to supervised contrastive learning, where multiple samples are known to belong to the same class. Therefore, we replaced the InfoNCE loss with the SupCon loss function.

## Methods

### Prior knowledge of histopathological images

Pathological prior knowledge includes the similarity of cellular and tissue morphology across different regions, as well as the spatial adjacency of areas belonging to the similar tissue type. Histopathological images are high-resolution, and detailed analysis of tissue morphology typically requires image segmentation. A WSI can be divided into tens of thousands of small patches. For non-diffuse cancers, similar tissues tend to cluster together, meaning that patches surrounding a particular patch usually belong to the same tissue type. This information serves as the pathological prior knowledge that we aim to incorporate into the self-supervised learning method, specifically the spatial adjacency of patches from the similar tissue. Inspired by the setup of supervised contrastive learning, we introduced pathological prior knowledge and sampled large-sized image patches to construct datasets for self-supervised learning.

During data loading, multiple small patches are sampled from each large patch and fed into the self-supervised framework for training. For any given small patch, other small patches sampled from the same large image block are treated as positive samples, while patches from different large image blocks are treated as negative samples.

### Representation learning framework

Our method is structurally based on existing SSL frameworks, but it is modified specifically for pathological images. In each training iteration, we begin by sampling multiple images from a batch of input data. We then apply two rounds of data augmentation to create two versions of the batch, which are subsequently passed through an encoder network to generate normalized embeddings. During training, these embeddings are further processed through a projection network, though this network will be discarded in downstream tasks. The loss is calculated using supervised contrastive loss (SupCon) based on the output of the projection network.

**Fig. 1:**
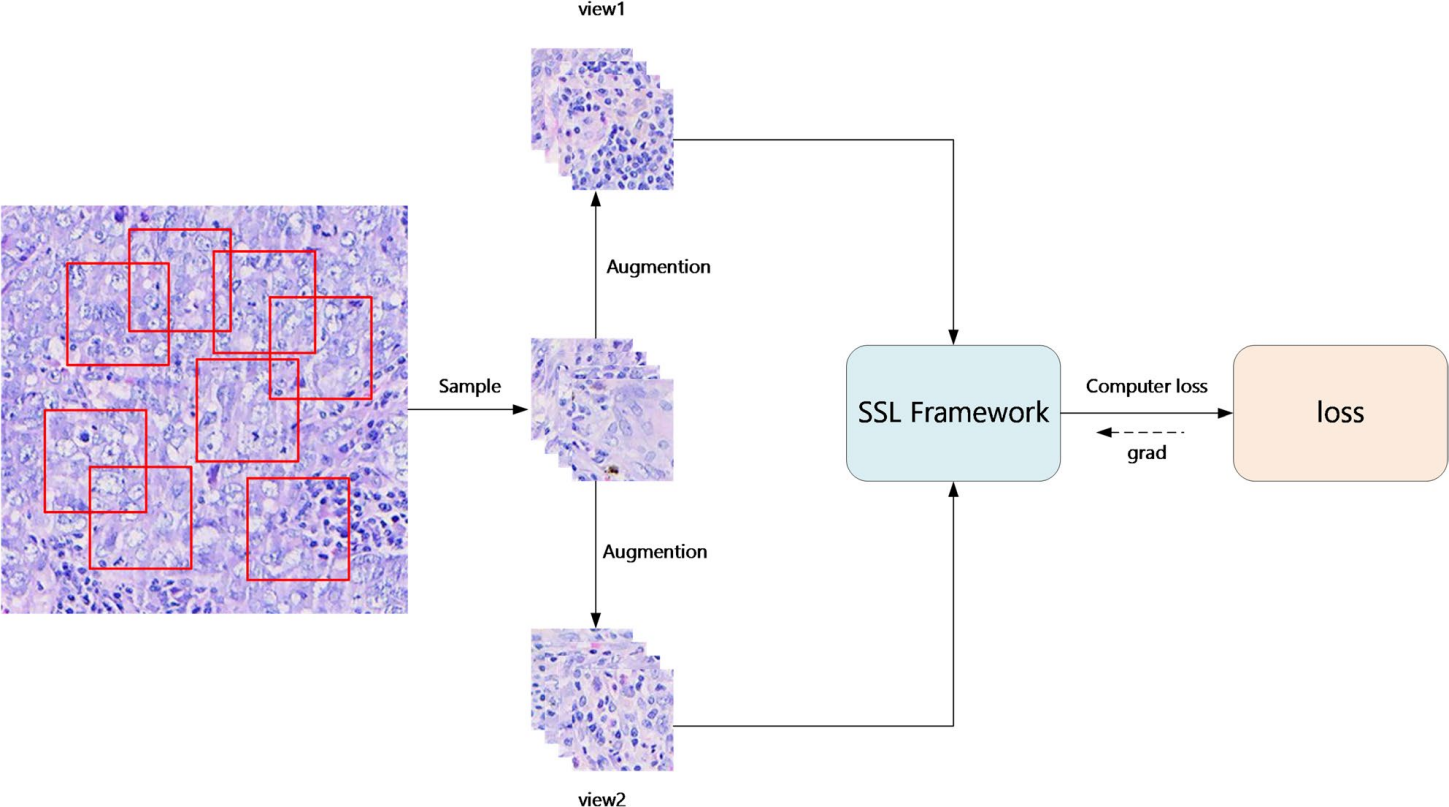
The framework of SSL with SP method

Our framework (Fig. 1) consists of the following key components:

Data Sampling Module, $\:Sam(\cdot)$. For each large input patch $\:X$, we randomly sample multiple smaller patches x=$\:Sam\left(X\right)$, For any small patch, other small patches sampled from the same large patch are treated as positive samples, while those from different large patches are considered negative samples. Therefore, within a single batch of a training iteration, each small patch has multiple positive and negative samples within the batch.

Data Augmentation module, $\:Aug\left(\cdot\:\right)$. For each input sample, $\:x$, we generate two random augmentations, $\:\sim x=Aug\left(x\right)$. Each augmented version represents a different view of the data and contains a subset of the information from the original sample. Different augmentations of the same image are considered similar and thus treated as positive pairs.

SSL Framework, $\:SSL\left(\cdot\:\right)$. This includes the encoder $\:Enc\left(\cdot\:\right)$ and the projection layer $\:Proj\left(\cdot\:\right)$. The framework learns from the input samples $\sim\,x$ to train an encoder $\:Enc\left(\cdot\:\right)$ with strong feature extraction capabilities.

Based on the above description, for $\:N$ large image patches as input, the sampling module generates $\:M\times\:N$ small patches. After processing through the data augmentation module, $\:2\times\:M\times\:N$ augmented images are obtained. These augmented images are then used as input to the self-supervised learning module.

For any given augmented image fed into the self-supervised module, other augmented images derived from the same large image patch are considered positive samples, totaling $\:(2\times\:M-1)$. Augmented images sampled from different large image patches are treated as negative samples, totaling $\:2\times\:M\times\:(N-1)$.

### Loss function

The supervised contrastive loss [25] for a given batch is formulated as follows:

$$\:\mathcal{L}=\sum\:_{i\in\:I}{\mathcal{L}}_{i}=\sum\:_{i\in\:I}\frac{-1}{\left|P\left(i\right)\right|}\sum\:_{p\in\:P\left(i\right)}\mathrm{log}\frac{exp\left({z}_{i}\cdot\:{z}_{p}/\tau\:\right)}{\sum\:_{a\in\:A\left(i\right)}exp\left({z}_{i}\cdot\:{z}_{a}/\tau\:\right)}$$

In our framework, let $\:z$ denote the self-supervised representations, and “$\:\cdot\:$” denote the inner product. The scalar $\:\tau\:\in\:{R}^{+}$is a temperature parameter, and $\:A\left(i\right)\equiv\:I\backslash\:i$. The index $\:i$ is referred to as the anchor. The set $\:P\left(i\right)\equiv\:\left\{p\in\:A\left(i\right):\stackrel{\sim}{{y}_{p}}=\stackrel{\sim}{{y}_{i}}\right\}$ contains the indices of all positive samples in the multi-view batch that are different from i, and $\:\left|\left.P\left(i\right)\right|\right.$is its cardinality. Other indices $\:\left\{k\in\:A\left(i\right)\backslash\:P\left(i\right)\right\}$are referred to as negative samples. $\:I$ is the set of all sample indices.

**Fig. 2:**
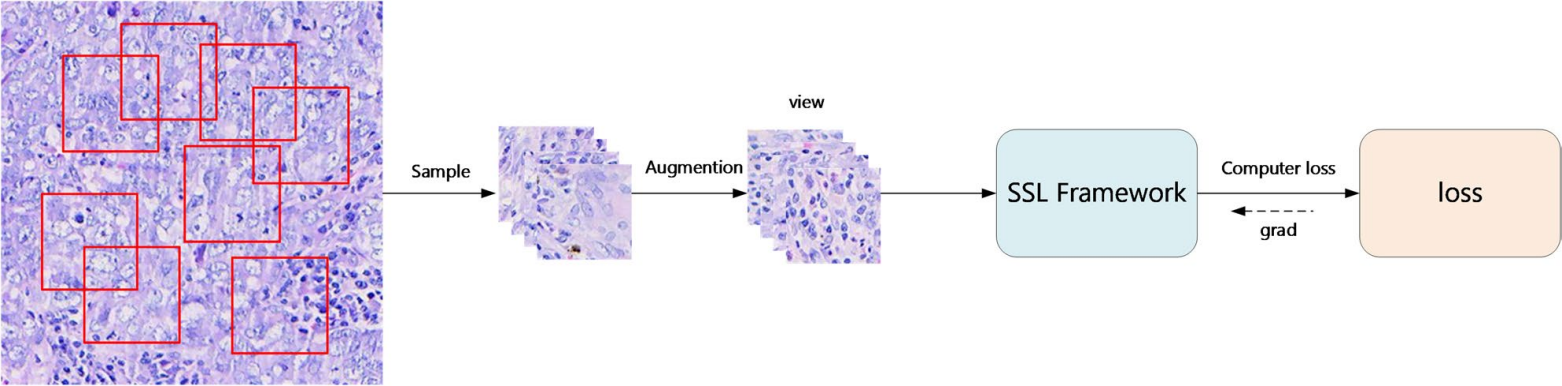
The framework of SinCLR

### Single-Branch Self-Supervised learning

Unlike traditional self-supervised learning (SSL), which typically relies on a dual-branch architecture to obtain positive sample pairs, our approach leverages prior knowledge of spatial adjacency of similar tissues from histopathological images. Specifically, by sampling adjacent small patches from the same large image patch and assuming that patches within the same large region are positive pairs, multiple positive pairs can be formed within a single batch of images. Therefore, we propose a single-branch SSL framework named SinCLR (single-branch framework for contrastive learning of visual representations), as illustrated in Fig. 2.

In the framework described in Representation learning framework, each batch of images undergoes two data augmentations, which are then fed into the model to extract features, forming a dual-branch structure for self-supervised learning. In contrast, SinCLR performs only one data augmentation and feeds it into the model to extract features, forming a single-branch structure for self-supervised learning. The loss function used in SinCLR remains consistent with that described in Loss function.

Based on the above description, for $\:N\:$large image patches as input, the sampling module generates $\:M\times\:N$ small patches. After processing through the data augmentation module, $\:M\times\:N$ augmented images are obtained. These augmented images are then used as input for the self-supervised learning module.

For any given augmented image fed into the self-supervised module, other augmented images derived from the same large image patch are considered positive samples, totaling $\:(M-1)$. Augmented images sampled from different large image patches are treated as negative samples, totaling $\:M\times\:(N-1)$.

## Experiment

In our experiments, we evaluate the performance of the proposed method on a WSIs dataset of lung squamous cell carcinoma (LSCC) from Fujian Cancer Hospital. The dataset consists of 65 LSCC slides from 65 patients, with no patient-level overlap. We divided the dataset into 37 slides and 28 slides.

To construct the dataset required for the downstream classification task, anexperienced pathologists independently annotated key areas of the slides, including tumor regions, stroma, epithelium, and areas of necrosis. The pathologist used standard histological criteria to guide the annotations. After the initial annotation, the data were reviewed by a senior pathologist to ensure consistency and resolve any potential discrepancies.

Briefly, histopathological slides were scanned on a Motic Digital Slide Scanner and analyzed using motic digital scanner assistant software, Motic VM 3.0. Under 100x magnification, we selected tumor bed areas(including the tumour epithelium and intratumour stroma) except for necrosis and blood vessels, and then calculated the selected areas by Image J software(ImageJ; National Institute of Health; http://rsbweb.nih.gov/ij).

After annotation, to extract representative image patches from the WSIs, we first remove the background using the Otsu method [30]. Subsequently, we apply a sliding window technique to generate 128 × 128 pixelspatches from the tissue regions of the slides at 20x magnification (0.5 μm/pixel). These patches were no overlap.

In summary, The downstream classification dataset, referred to as Dataset 1, contains four tissue categories: tumor, stroma, epithelium, and necrosis. Image patches for the training and validation sets were sampled from the 37 slides at an 8:2 ratio, while the test set patches were sampled from the remaining 28 slides.

To construct the pretraining datasets for the SSL methods, we removed the background using the Otsu method. Subsequently, a sliding window technique was applied to the tissue regions to generate image patches of two sizes: 128× 128 pixels and 512 × 512 pixels. The 128 × 128 pixels patches were used to create Dataset 2 for pretraining baseline SSL methods, whereas the 512 × 512 pixels patches were used to construct Dataset 3 for pretraining the proposed SP framework. To ensure that pretraining did not influence the downstream classification task, both Dataset 2 and Dataset 3 were sampled from the 37 slides. The detailed statistics of each dataset are summarized in Table 1.

**Table 1:** Detailed information for each dataset

Total WSIs		65 (from 65 LSCC patients)		
Slides splitting		37 slides		28 slides
Dataset 1	Application	training	validation	test
	Image number	65,806	16,452	49,802
	Image size	128 × 128 pixels		
	Source	annotated regions of WSIs		
Dataset 2	Application	pretraining baseline SSL methods		/
	Image number	114,000		
	Image size	128 × 128 pixels		
	Source	patches sampled from tissue regions of WSIs		
Dataset 3	Application	pretraining the proposed SP framework		/
	Image number	5,311		
	Image size	512 × 512 pixels		
	Source	patches sampled from tissue regions of WSIs		

In our study, we integrate the proposed method with several widely used SSL frameworks, including SimCLR, SimSiam, and MoCo-v3 [31], and compare them against both supervised models and the original SSL frameworks as baselines. All SSL methods and supervised models utilize a ResNet18 [32] backbone with a batch size of 128. The SSL models are trained for 200 epochs, while the supervised models are trained for 50 epochs using an early stopping strategy. To ensure a reliable comparison, the hyperparameters for training are set as consistently as possible. Detailed hyperparameter settings are provided in the supplementary file.

We evaluated our method on a four-class classification task for tumor regions, stroma, epithelium, and areas of necrosis, conducting fine-tuning and linear evaluation on the annotated dataset. The evaluation metrics included accuracy (Acc), area under the curve (AUC), and F1 score.

### Fine-tuning

Table 2 presents the performance of various methods under fine-tuning. The results show that pre-trained models significantly outperform models trained from scratch, with SSL pre-trained models achieving notably better results than those pre-trained on ImageNet. Among the contrastive learning frameworks, our proposed method demonstrates superior performance compared to the baseline methods. Specifically, SPSimCLR shows the greatest improvement over SimCLR (F1 score: 0.9132 vs. 0.9067), and SPMoCo-v3 also outperforms MoCo-v3 (F1 score: 0.9133 vs. 0.9088). However, within the knowledge distillation-based methods, SPSimSiam performs slightly worse than the original SimSiam method and even lags behind the models pre-trained on ImageNet. Notably, our proposed SinCLR shows a slight performance decrease compared with our dual-branch methods such as SPSimCLR, but achieves performance comparable to baseline methods such as SimCLR and SimSiam, while reducing memory consumption and training time by one-third (memory usage: 6000 MB vs. 9000 MB; training time: 16 h vs. 24 h). The performance of these methods is also illustrated by the ROC curve in Supplementary Fig. 1.

**Table 2:** The fine-tuning performance of different methods on the lung squamous cell carcinoma dataset

Method	Acc	Auc	F1 score
Ramdom	0.9128	0.9744	0.8789
Pretrained	0.9242	0.9824	0.8942
SimCLR	0.9328	0.9871	0.9067
Simsiam	0.9322	0.9862	0.9053
MoCo-v3	0.9339	0.9890	0.9088
SPSimCLR	0.9371	0.9903	0.9132
SPSimsiam	0.9227	0.9855	0.8915
SPMoCo-v3	0.9366	0.9901	0.9133
SinCLR	0.9323	0.9892	0.9074

### Linear evaluation

For linear evaluation, we freeze the backbone of the models and train only the classification head. Table 3 presents the performance of each method under linear evaluation. The results demonstrate that SPSimCLR(F1 score: 0.9082) and SPMoCo-v3(F1 score: 0.9060) significantly outperform SimCLR(F1 score: 0.8978) and MoCo-v3(F1 score: 0.8942), while SPSimsiam still performs the worst. SinCLR(F1 score: 0.9021) achieves better results than the baseline methods, indicating that models trained with our method have superior feature extraction capabilities. The performance of these methods is also illustrated by the ROC curve in Supplementary Fig. 2.

**Table 3:** The linear evaluation results of different methods on the lung squamous cell carcinoma dataset

Method	Acc	Auc	F1 score
Pretrained	0.8825	0.9582	0.8391
SimCLR	0.9258	0.9841	0.8978
Simsiam	0.9214	0.9887	0.8946
MoCo-v3	0.9230	0.9830	0.8942
SPSimCLR	0.9326	0.9875	0.9082
SPSimsiam	0.8564	0.9597	0.7881
SPMoCo-v3	0.9303	0.9874	0.9060
SinCLR	0.9288	0.9867	0.9021

### Ablation study

To assess the method’s applicability, we performed separate ablation experiments focusing on sampling methods and image content.

**Fig. 3:**
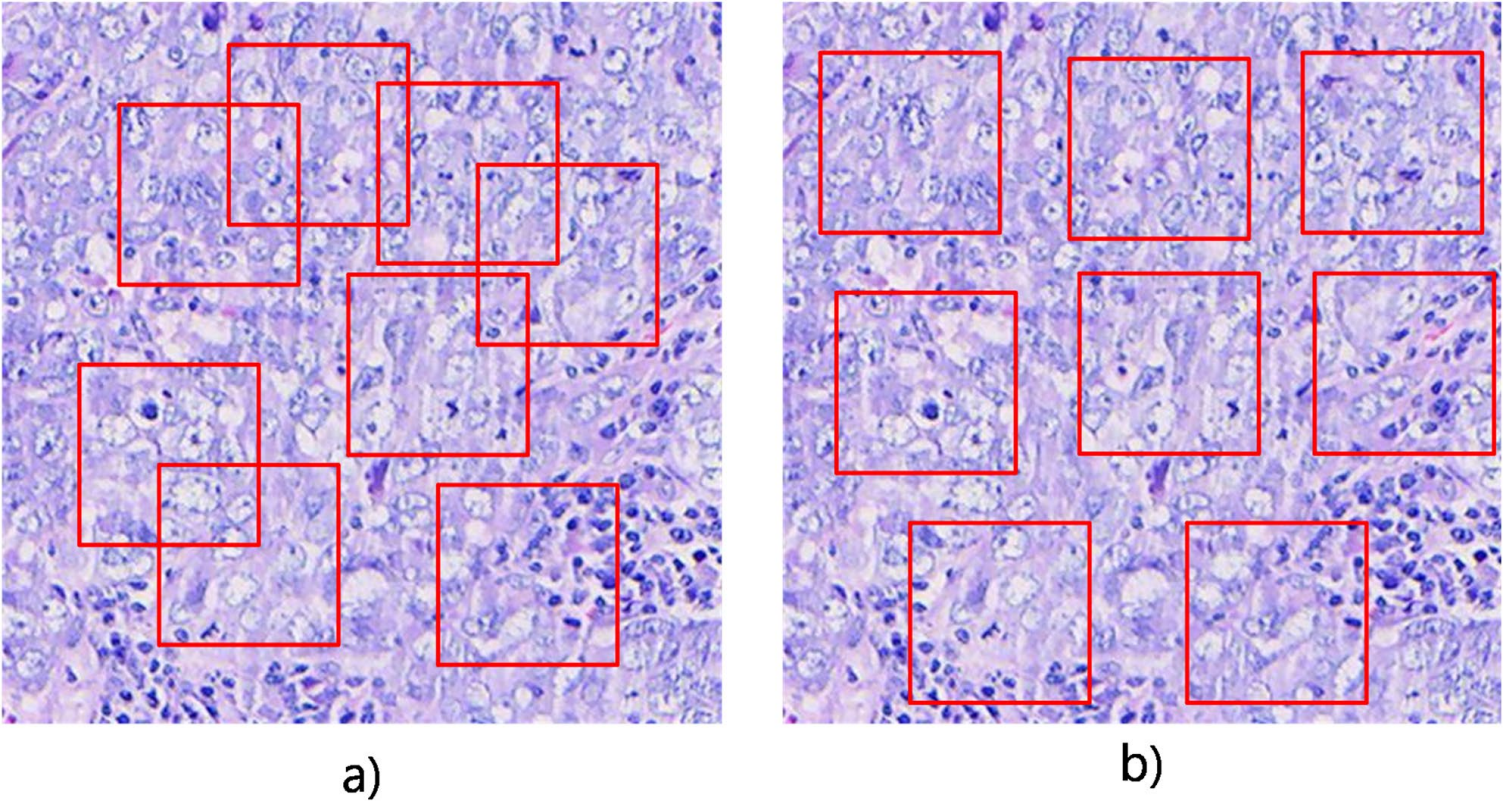
Two different sampling methods, **a**) Overlapping sampling, **b**) Non-overlapping sampling

#### Sampling strategy

Different image sampling strategies are employed to generate positive samples, which may impact the model’s performance. As shown in Fig. 3, two sampling methods were examined: (a) Overlapping Sampling and (b) Non-overlapping Sampling.

In the Overlapping Sampling method, samples are randomly selected within each image, allowing for potential overlap among positive samples. Conversely, the Non-overlapping Sampling method imposes strict constraints, preventing positive samples from overlapping while allowing them to be adjacent.

**Table 4:** The fine-tuning results of different sampling methods on the lung squamous cell carcinoma dataset

	Method	Acc	Auc	F1 score
Overlapping	SPSimCLR	0.9371	0.9903	0.9132
	SPMoCo-v3	0.9366	0.9901	0.9133
	SinCLR	0.9323	0.9892	0.9074
Non-overlapping	SPSimCLR	0.9355	0.9903	0.9106
	SPMoCo-v3	0.9350	0.9897	0.9107
	SinCLR	0.9314	0.9886	0.9050

Table 4 and Supplementary Table 3 show that models trained with overlapping sampling achieve slightly better performance than those trained with non-overlapping sampling (F1 score: 0.9132 vs. 0.9106, 0.9133 vs. 0.9107 and 0.9074 vs. 0.9050). This may be attributed to overlapping sampling providing similar information among samples due to the shared regions, which facilitates the convergence of positive samples. In contrast, non-overlapping sampling relies solely on the model to drive the convergence of positive samples, which may lead to the learning of partial noise information. The performance of these methods is also illustrated by the ROC curve in Supplementary Figs. 3 and 4.

#### Image content

Our method is based on the assumption that similar tissues tend to cluster within large patches to create positive sample pairs. To assess the impact of heterogeneous tissues within these patches, we sampled multiple points along annotated curves and extracted large patches centered at these points. This approach ensures that the resulting patches are likely to contain multiple tissue types. We then trained models on this new dataset and compared their performance with models trained on datasets created using sliding window segmentation and uniform sampling.

**Table 5:** The fine-tuning results for images with different tissue types on the lung squamous cell carcinoma dataset

	Method	Acc	Auc	F1 score
Single tissue	SPSimCLR	0.9371	0.9903	0.9132
	SPMoCo-v3	0.9366	0.9901	0.9133
	SinCLR	0.9323	0.9892	0.9074
Multiple tissues	SPSimCLR	0.9302	0.9885	0.9032
	SPMoCo-v3	0.9263	0.9868	0.8984
	SinCLR	0.9244	0.9859	0.8948

Table 5 and Supplementary Table 4 reveal that the presence of multiple tissue types in the training samples significantly affects model performance, with an overall decrease in the F1 score (F1 score: 0.9132 vs. 0.9032, 0.9133 vs. 0.8984 and 0.9074 vs. 0.8948). The reason behind this might be attributed to the assumption of contrastive learning that positive sample pairs have similar representations. However, when big patches contain multiple tissue types, the generated positive sample pairs belong to different categories, contradicting the method’s assumption and introducing substantial noise. Consequently, during model training, different tissue representations are forced to converge, which diminishes the model’s representational capacity. Therefore, our method is not suitable for diffuse cancers. The performance of these methods is also illustrated by the ROC curve in Supplementary Figs. 5 and 6.

This Ablation Study also reveal that at tissue boundaries, slight morphological variations may cause adjacent patches to belong to different tissue types, leading to mislabeling of positive samples. When large image patches are sampled randomly under normal conditions, it is unavoidable to include boundary patches, which may affect the performance of our method. Currently, there is no perfect solution for this issue, representing a limitation of the approach.

## Discussion

### Results analysis

Based on the comprehensive analysis of fine-tuning and linear evaluation results, leveraging the prior knowledge that similar tissues tend to cluster, increasing the number of positive samples in the contrastive learning framework improves performance. In contrast, methods based on knowledge distillation show a decline in model’s performance. This can be explained by the nature of contrastive learning, which optimizes sample representations by contrasting positive and negative pairs, thereby promoting the clustering of positive samples while separating negative ones in the feature space. Our method enhances performance by increasing the number of positive samples, thereby preventing samples belonging to the same class from being mistakenly categorized as negative sample pairs and causing separation. However, in knowledge distillation-based learning, an excessive number of positive samples can lead to the convergence of all sample representations, potentially resulting in model collapse.

**Fig. 4:**
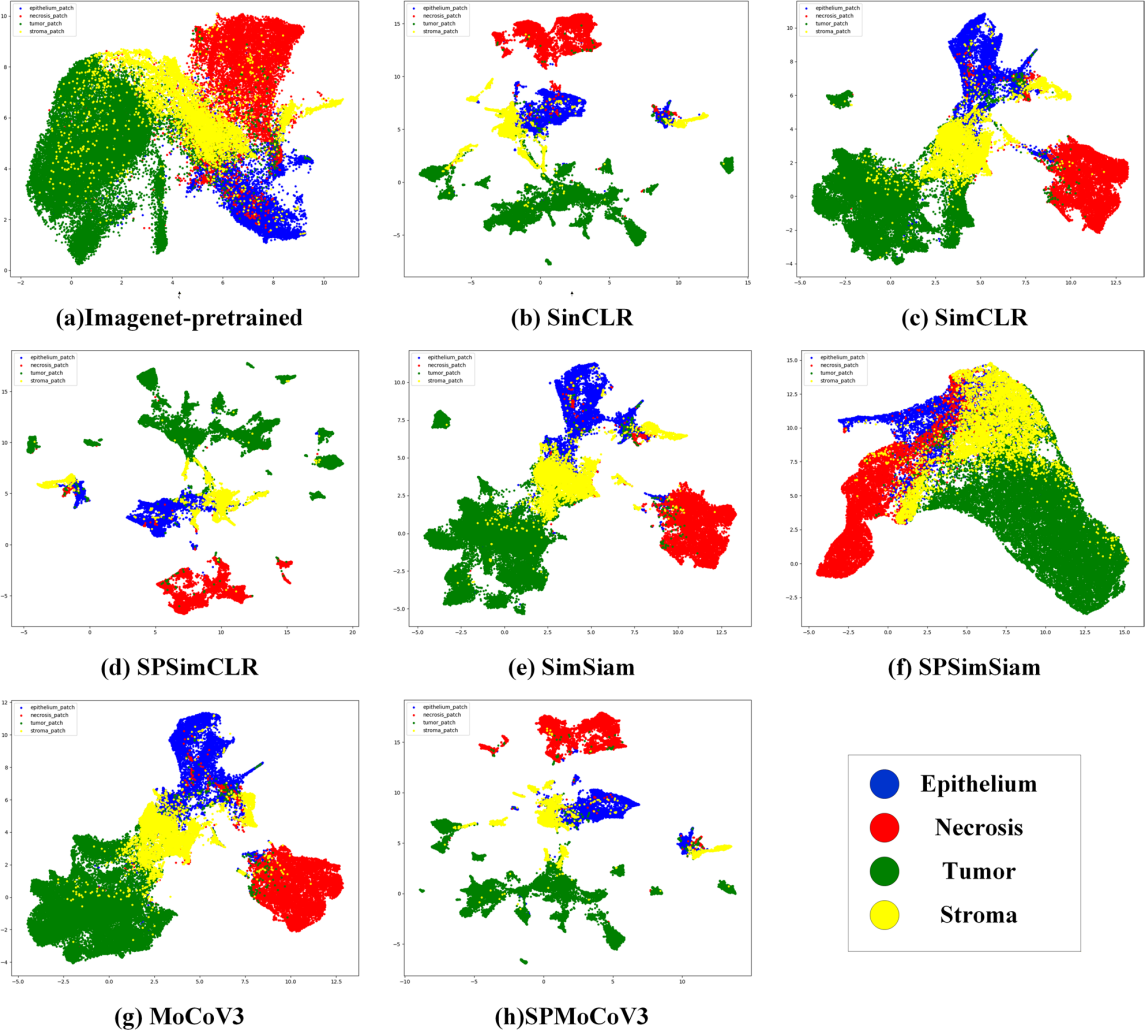
Visualization of eight embedding spaces using UMAP. Instances in the test set are color-coded according to their ground-truth tissue labels: Blue for Epithelium, Red for Necrosis, Green for Tumor, and Yellow for Stroma

We removed the classification head from the model, retained the backbone, and input the data into the unsupervisedly trained model to obtain sample representations. These representations were then reduced to two-dimensional space using the UMAP method [33] and visualized as scatter plots. Figure 4 shows the sample spaces produced by each method. Consistent with the evaluation tasks, the ImageNet pre-trained model (a) and the model pre-trained with SPSimsiam (f) exhibit clusters of various class samples, with substantial overlap of samples from different classes within each cluster. In contrast, the sample spaces of models pre-trained with other methods show well-separated clusters for each class.

Comparing SimCLR (c) with SPSimCLR (d) and MoCo-v3 (e) with SPMoCo-v3 (f), we observed that the SP method leads to more pronounced separation between clusters of different class samples. This further confirms that the SP method enhances contrastive learning performance in the domain of pathology images. The simplified model, SinCLR (b), also demonstrates clear separation between clusters of different class samples, indicating its performance is comparable to that of traditional contrastive learning methods.

### Results visualization

Based on the comprehensive results, SPSimCLR demonstrates the best performance. under fine-tuning. Therefore, we selected SPSimCLR as the final model for WSIs analysis.

**Fig. 5:**
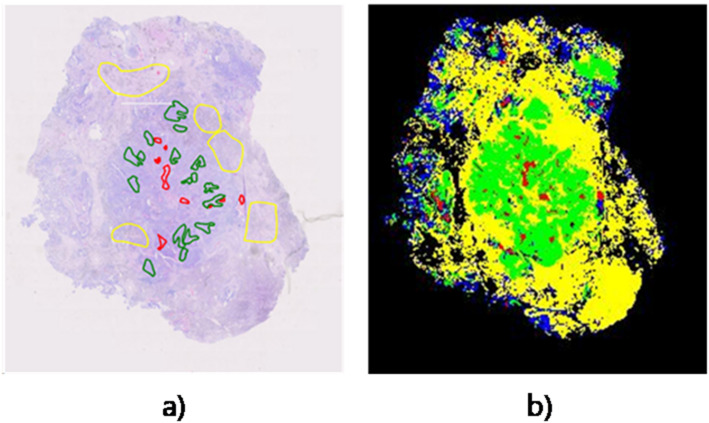
Example of WSI analysis. **a** Partial ground truth annotations showing a subset of manually labeled regions. **b** Model prediction results

We apply Ostu’s method to filter out the background and use sliding windows to extract patches. These patches are subsequently fed into the model for prediction. Based on the prediction results, we color the masks to obtain the analysis results of the WSIs. Figure 5 shows that the model can accurately identify small amounts of necrotic tissue, demonstrating its effectiveness in assisting pathologists with WSIs analysis.

### Experimental limitations

The experiments conducted in this study were validated on only a single-center dataset with a limited sample size, which is one of the limitations of our work. Validation on additional external datasets would further demonstrate the generalizability of the proposed method—for example, evaluating on the HER2 Warwick Dataset as done by Chyrmang Genevieve et al. [34], or incorporating TCGA cohorts as performed by other researchers [35, 36].

However, applying the model to multi-center or cross-cohort datasets may introduce several challenges. Differences in staining protocols, scanning equipment, and slide preparation across centers may lead to domain shifts, which require additional domain adaptation techniques or color normalization strategies to mitigate. Following the dataset construction procedure described in Experiment, some histopathological images lack annotations and need manual labeling by experienced pathologists. We also need to perform background removal using the Otsu method and apply sliding-window sampling to extract tissue patches before entering the validation stage. The processes of data collection, annotation, and dataset construction are time-consuming. In the future, we plan to validate our method on more external datasets and continue improving the proposed framework.

## Conclusion

In this study, we propose the SP technique, specifically designed for non-diffuse cancer pathology images. By integrating prior knowledge of pathology images that some similar tissues are spatially adjacent with contrastive learning methods, the SP technique enhances model performance in distinguishing histological categories in LSCC. Additionally, we introduced SinCLR, a single-branch contrastive learning framework that performs similarity comparisons within the same branch. SinCLR enhances representation capability while significantly reduceing memory usage and training time compared to dual-branch methods, achieving performance comparable to the original dual-branch methods. However, this study has certain limitations. The method may be affected by tissue at the boundaries, which could impact model performance. To address this, we are considering introducing a learnable network that maps large image patches to weighting factors to reduce such effects. Additionally, we have not performed validation on external datasets. In future work, we plan to validate our method on independent cohorts to further strengthen the robustness of our findings.

## Supplementary Information

Supplementary Material 1.

## Data Availability

The data that support the results of this study are available from the corresponding author on reasonable request.
