# Supplementary material for: A contrastive learning method integrating pathological prior information for effective differentiation of histological categories in lung squamous cell carcinoma
Source: BMC Cancer. 2025 Dec 17;26:114. doi: 10.1186/s12885-025-15459-0 (PMC12831444; doi:10.1186/s12885-025-15459-0)
Supplement: Supplementary file 1 — Supplementary Material 1. [file 12885_2025_15459_MOESM1_ESM.docx]

## Hyperparameter Settings

For SSL, the method utilizes ResNet18 as the backbone network with a batch size of 128, trained for 200 epochs. It employs the SGD optimizer with a momentum of 0.9, weight decay of 0, and adopts a 10-epoch warm-up followed by 190 epochs of cosine annealing learning rate strategy. The initial learning rate is set to 1e-4, and after the warm-up, it adjusts to 0.2. The model projection layer follows the settings in the original paper. The data augmentation is shown in Supplementary Table 1.

For supervised learning, ResNet18 serves as the backbone network with a batch size of 128. It employs an early stopping strategy, training for 50 epochs. Specifically, training halts if the validation set loss doesn't decrease for 10 consecutive epochs. It uses the SGD optimizer with a momentum of 0.9 and weight decay of 10e-4. The initial learning rate for fine-tuning is set to 1e-3 (while the randomly initialized model's initial learning rate is 0.01), and the initial learning rate for linear evaluation is set to 0.1. The learning rate undergoes a fixed decay of 10x at epochs 10 and 30. The data augmentation is shown in Supplementary Table 2.

## Supplementary Table and Figure

**Supplementary Table 1. Data augmentation policy used by SSL**

| Data augmentation | parameter | probability |
| --- | --- | --- |
| RandomResizedCrop | scale=(0.2, 1.0) | / |
| RandomColorJitter | (0.4, 0.4, 0.4, 0.1) | 0.8 |
| RandomGrayscale | / | 0.2 |
| RandomGaussianBlur | [0.1, 2.0] | 0.5 |
| RandomHorizontalFlip | / | 0.5 |
| Normalize | mean = [0.6684, 0.5115, 0.6791] std = [0.2521, 0.2875, 0.2100] | / |

**Supplementary Table 2. Data augmentation policy used by supervised learning**

| Data augmentation | parameter | probability |
| --- | --- | --- |
| RandomColorJitter | (0.25, 0.75, 0.25, 0.04) | 0.8 |
| RandomHorizontalFlip | / | 0.5 |
| Normalize | mean = [0.6684, 0.5115, 0.6791] std = [0.2521, 0.2875, 0.2100] | / |

**Supplementary Table 3. The linear evaluation results of different sampling methods on the lung squamous cell carcinoma dataset.**

|  | Method | Acc | Auc | F1 score |
| --- | --- | --- | --- | --- |
| Overlapping | SPSimCLR | 0.9326 | 0.9875 | 0.9082 |
|  | SPMoCo-v3 | 0.9303 | 0.9874 | 0.9060 |
|  | SinCLR | 0.9288 | 0.9867 | 0.9021 |
| Non-overlapping | SPSimCLR | 0.9325 | 0.9881 | 0.9084 |
|  | SPMoCo-v3 | 0.9308 | 0.9867 | 0.9059 |
|  | SinCLR | 0.9283 | 0.9859 | 0.9013 |

**Supplementary Table 4. The linear evaluation results for images with different tissue types on the lung squamous cell carcinoma dataset.**

|  | Method | Acc | Auc | F1 score |
| --- | --- | --- | --- | --- |
| Single tissue | SPSimCLR | 0.9326 | 0.9875 | 0.9082 |
|  | SPMoCo-v3 | 0.9303 | 0.9874 | 0.9060 |
|  | SinCLR | 0.9288 | 0.9867 | 0.9021 |
| Multiple tissues | SPSimCLR | 0.9262 | 0.9845 | 0.8971 |
|  | SPMoCo-v3 | 0.9199 | 0.9837 | 0.8894 |
|  | SinCLR | 0.9171 | 0.9815 | 0.8832 |


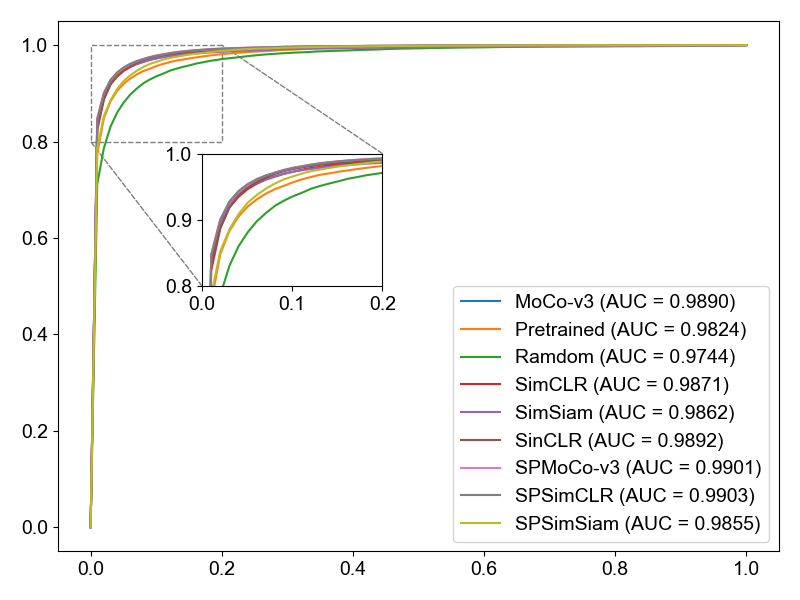


**Supplementary Figure 1.** The fine-tuning ROC curve of different methods on

the lung squamous cell carcinoma dataset.


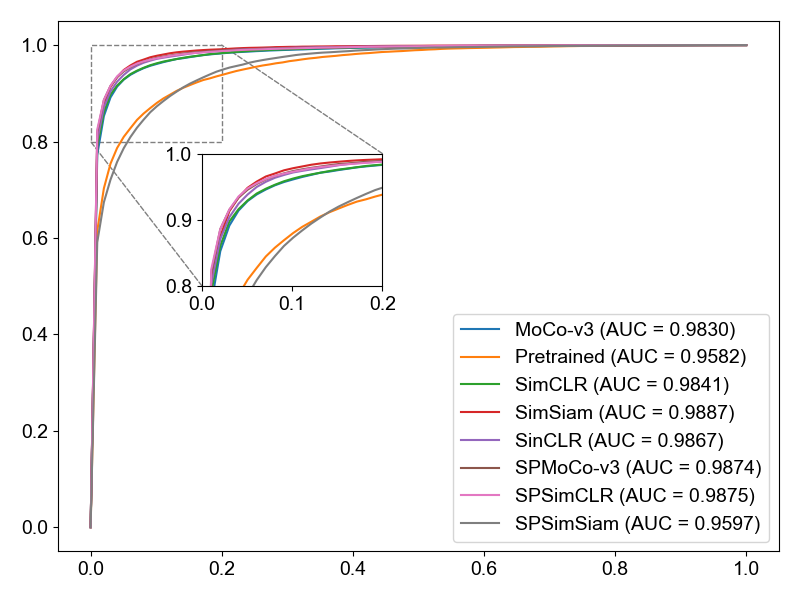


**Supplementary Figure 2.** The linear evaluation ROC curve of different methods on

the lung squamous cell carcinoma dataset.


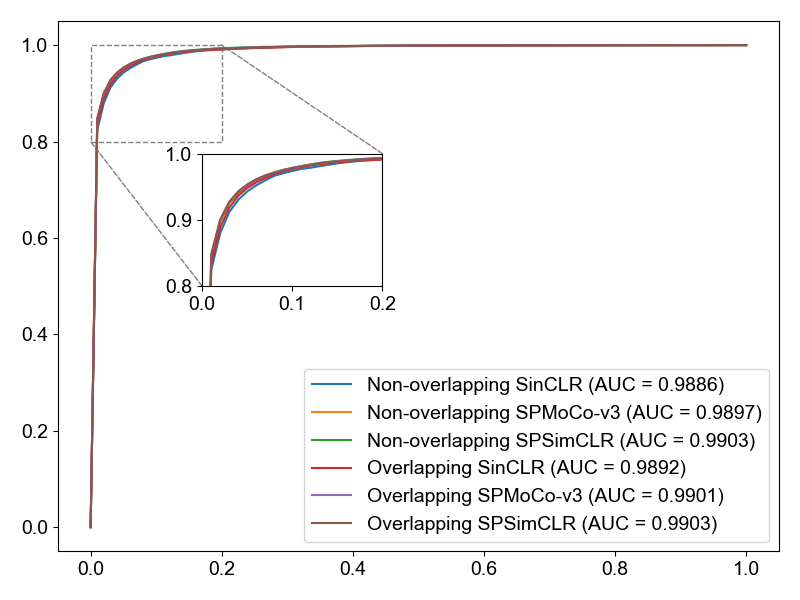


**Supplementary Figure 3.** The fine-tuning ROC curve of different sampling methods on the lung squamous cell carcinoma dataset.


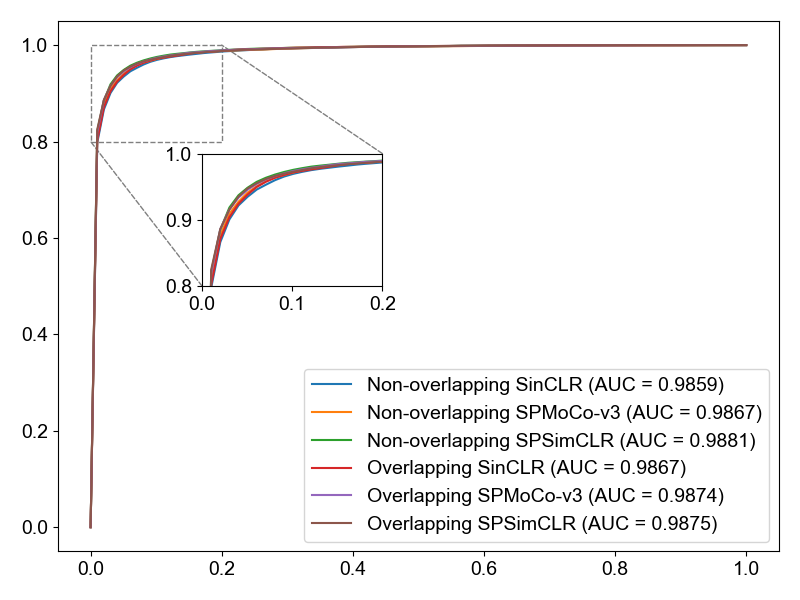


**Supplementary Figure 4.** The linear evaluation ROC curve of different sampling

methods on the lung squamous cell carcinoma dataset.


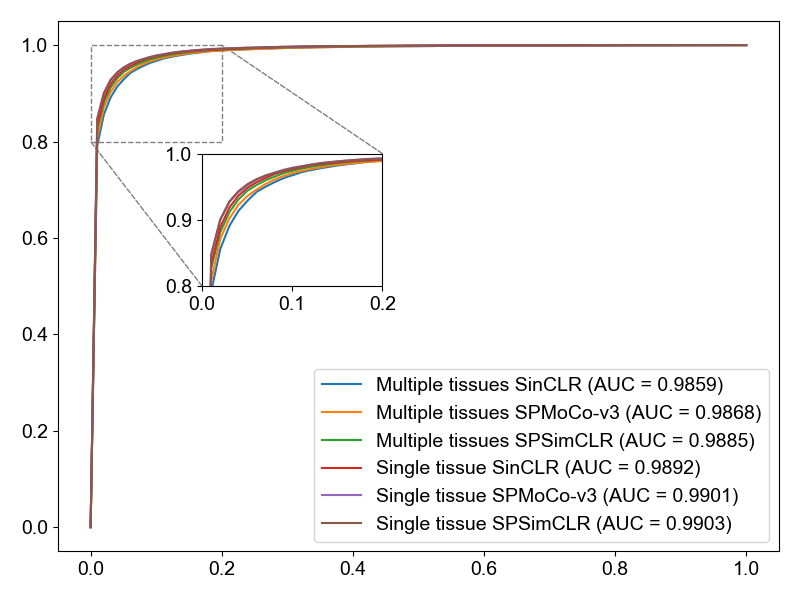


**Supplementary Figure 5.** The fine-tuning ROC curve for images with different tissue types on the lung squamous cell carcinoma dataset.


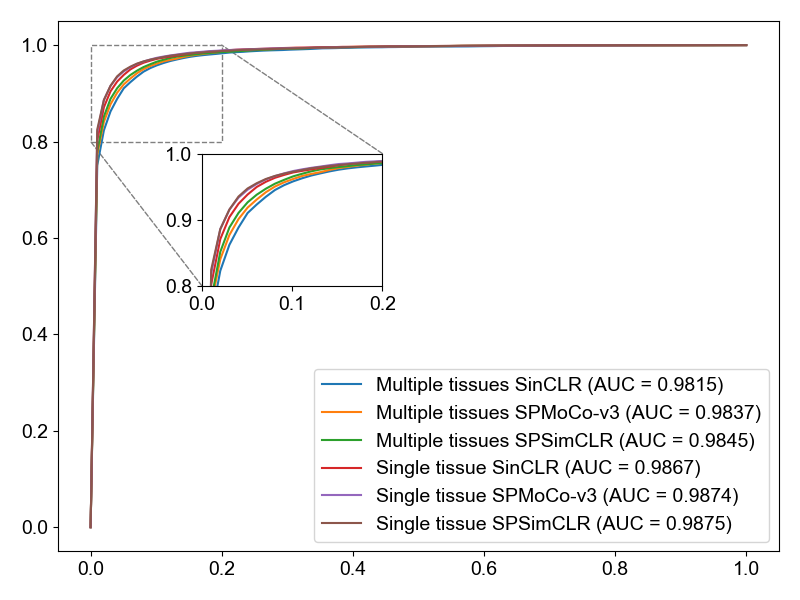


**Supplementary Figure 6.** The linear evaluation ROC curve for images with different tissue types on the lung squamous cell carcinoma dataset.

**Supplementary Figure 7.** Example of WSI analysis. (a) Partial ground truth annotations showing a subset of manually labeled regions. b) Model prediction results.
